# LEA polypeptide profiling of recalcitrant and orthodox legume seeds reveals ABI3-regulated LEA protein abundance linked to desiccation tolerance

**DOI:** 10.1093/jxb/ert274

**Published:** 2013-09-16

**Authors:** Julien Delahaie, Michaela Hundertmark, Jérôme Bove, Olivier Leprince, Hélène Rogniaux, Julia Buitink

**Affiliations:** ^1^Université d’Angers, UMR 1345 Institut de Recherche en Horticulture et Semences, SFR 4207 QUASAV, PRES L’UNAM, 49045 Angers, France; ^2^Agrocampus Ouest, UMR 1345 Institut de Recherche en Horticulture et Semences, SFR 4207 QUASAV, PRES L’UNAM, 49045 Angers, France; ^3^Institut National de la Recherche Agronomique, UR1268 Biopolymères, Interactions, Assemblages, Plate-forme Biopolymères-Biologie Structurale, 44316 Nantes, France; ^4^Institut National de la Recherche Agronomique, UMR 1345 Institut de Recherche en Horticulture et Semences, SFR 4207 QUASAV, PRES L’UNAM, 49045 Angers, France

**Keywords:** abi3, Castanospermum australe, desiccation tolerance, late embryogenesis abundant proteins, Medicago truncatula, proteomics, recalcitrant seed, RNAseq.

## Abstract

In contrast to orthodox seeds that acquire desiccation tolerance during maturation, recalcitrant seeds are unable to survive drying. These desiccation-sensitive seeds constitute an interesting model for comparative analysis with phylogenetically close species that are desiccation tolerant. Considering the importance of LEA (late embryogenesis abundant) proteins as protective molecules both in drought and in desiccation tolerance, the heat-stable proteome was characterized in cotyledons of the legume *Castanospermum australe* and it was compared with that of the orthodox model legume *Medicago truncatula*. RNA sequencing identified transcripts of 16 homologues out of 17 LEA genes for which polypeptides are detected in *M. truncatula* seeds. It is shown that for 12 LEA genes, polypeptides were either absent or strongly reduced in *C. australe* cotyledons compared with *M. truncatula* seeds. Instead, osmotically responsive, non-seed-specific dehydrins accumulated to high levels in the recalcitrant cotyledons compared with orthodox seeds. Next, *M. truncatula* mutants of the *ABSCISIC ACID INSENSITIVE3* (*ABI3*) gene were characterized. Mature *Mtabi3* seeds were found to be desiccation sensitive when dried below a critical water content of 0.4g H_2_O g DW^–1^. Characterization of the LEA proteome of the *Mtabi3* seeds revealed a subset of LEA proteins with severely reduced abundance that were also found to be reduced or absent in *C. australe* cotyledons. Transcripts of these genes were indeed shown to be *ABI3* responsive. The results highlight those LEA proteins that are critical to desiccation tolerance and suggest that comparable regulatory pathways responsible for their accumulation are missing in both desiccation-sensitive genotypes, revealing new insights into the mechanistic basis of the recalcitrant trait in seeds.

## Introduction

Global agriculture and the conservation of plant biodiversity rely on seeds and their ability to be stored for long periods of time in dedicated national and international storage facilities (Li and Pritchard, 2009; Walters *et al.*, 2013). The terms ‘orthodox’ and ‘recalcitrant’ are used to describe the storage behaviour of seeds. Orthodox seeds undergo maturation drying and are shed from the parent plant at low moisture contents. During maturation, they acquire desiccation tolerance, allowing them to be dried to moisture contents in the range of 1–5% without irreversible damage. Because of this ability, seeds can be stored for long periods in cold and dry vaults. Recalcitrant seeds, on the other hand, do not undergo maturation drying, and are shed at relatively high moisture contents. Such seeds are highly susceptible to desiccation injury, and thus are not storable under conditions suitable for orthodox seeds (reviewed in Farnsworth, 2000; Berjak and Pammenter, 2008; Li and Pritchard, 2009). The mechanisms by which recalcitrant seeds lose viability during drying and/or storage are not well understood, which poses a challenge to determine appropriate measures to better conserve these species.

In orthodox seeds, isolation and analysis of viviparous mutants and loss-of-function mutants impaired in embryogenesis and seed maturation resulted in the identification of master seed development regulator loci *lec1* and *abi3*, regulating partial and redundant desiccation tolerance (Ooms *et al.*, 1993; Parcy *et al.*, 1997; To *et al.*, 2006). A third regulator, *FUSCA3*, appears to control seed longevity (Tiedemann *et al.*, 2008). In *Arabidopsis* and maize, some of the target genes of these activators are genes proposed to have a protective role in desiccation tolerance, such as small heat shock protein genes, genes with antioxidant functions, as well as late embryogenesis abundant (LEA) genes (Kotak *et al.*, 2007; Bies-Etheve *et al.*, 2008; Mönke *et al.*, 2012).

LEA proteins are small hydrophilic, largely unstructured and thermostable proteins that are synthesized in orthodox seeds during mid- to late maturation and in vegetative tissues upon osmotic stress. They are thought to have a range of protective functions against desiccation with different efficiencies, including ion binding, antioxidant activity, hydration buffering, and membrane and protein stabilization (Tunnacliffe and Wise, 2007; Battaglia *et al.*, 2008; Amara *et al.*, 2012). Evidence of an *in vivo* role for these proteins in seed desiccation comes from *Arabidopsis thaliana em6*-deficient mutants that show defects in maturation drying (Manfre *et al.*, 2009). A recent study in *A. thaliana* showed that down-regulation of seed-specific dehydrins (one of the LEA families) reduced seed survival in the dry state, although seeds did acquire desiccation tolerance (Hundertmark *et al.*, 2011). However, the precise role of LEA proteins in seed desiccation tolerance remains to be ascertained for the vast majority of them. Genomic studies to date have identified a large number of LEA genes whose expression is restricted to seed tissues and/or up-regulated in response to biotic and abiotic stress in vegetative tissues (Illing *et al.*, 2005; Hundertmark and Hincha, 2008; Amara *et al.*, 2012; Chatelain *et al.*, 2012). Proteomic studies demonstrate that a subset of polypeptides accumulate during the acquisition of desiccation tolerance and/or longevity in orthodox seeds (Boudet *et al.*, 2006; Buitink *et al.*, 2006; Chatelain *et al.*, 2012). In desiccation-sensitive seeds of *Arabidopsis* mutants, transcript levels of several LEA genes were reduced, whereas other increased (e.g. dehydrins) (Bies-Etheve *et al.*, 2008).

The situation regarding the occurrence and role of LEAs in recalcitrant seeds is ambiguous. Work has been constrained to detect members of the dehydrin family and showed that they are present in a range of species from different habitats, while apparently being absent from others. Several studies reported the presence of dehydrins in recalcitrant species of temperate origin, whereas these proteins could not be detected in some highly desiccation-sensitive seeds from certain tropical species (Finch-Savage *et al.*, 1994; Farrant *et al.*, 1996; Han *et al.*, 1997; Hinniger *et al.*, 2006; Panza *et al.*, 2007; Sunderlikova *et al.*, 2009; Ismail *et al.*, 2010; Lee *et al.*, 2012). In stored recalcitrant seeds of *Quercus robur* L., dehydrin mRNA can also be induced by abscisic acid (ABA) and limited drying treatments (Finch-Savage *et al.*, 1994). Whereas the presence/absence of dehydrins cannot explain the recalcitrant behaviour of the species studied to date, several other families of LEA proteins exist in orthodox seeds that have not been studied in recalcitrant seeds.

A comparative analysis of recalcitrant and orthodox seed development is an interesting alternative to identify mechanisms involved in desiccation tolerance, especially if closely related species are compared (Kermode, 1997; Oliver *et al.*, 2011). Recently, the comparison of the metabolomic responses of drying leaves of two closely related grass species (sister group contrast), one being desiccation tolerant and the other desiccation sensitive, highlighted the metabolic predispositions associated with desiccation tolerance (Oliver *et al.*, 2011). In this study, a recalcitrant seed species of the *Papilionaceae* subfamily was characterized to allow comparison with previous studies on orthodox seeds of the model legume *Medicago truncatula*. The phylogenetically closest recalcitrant species for which seeds can currently be obtained is *Castanospermum australe* A.Cunn ex Hook. (Doyle, 1995). *Castanospermum australe* is a tropical tree native of east Australia and now implanted in South Africa and Sri Lanka. In the seeds of this species, dehydrins were detected by western blot analysis (Han *et al.*, 1997). The absence of a sequenced genome of this species impedes the thorough molecular comparison of the entire LEA proteome with orthodox seeds. Thus, high-throughput sequencing technology was used to obtain, assemble, and annotate the transcriptome of these recalcitrant seeds. Whereas transcripts could be detected in *C. australe* for most LEA genes that are present in the desiccation-tolerant *M. truncatula* seeds, a comparative analysis of the LEA proteome profiles revealed that abundance for a number of seed-specific LEA proteins was severely affected in the recalcitrant seeds. In contrast, homologues of several dehydrins that are expressed in seedlings or non-seed tissues of *M. truncatula* submitted to osmotic stress accumulated to high levels in *C. australe* seeds. Comparison of the LEA proteome with desiccation-sensitive *abi3* mutants of *M. truncatula* showed a comparable reduction of a number of seed-specific LEA proteins.

## Materials and methods

### Plant material and treatments

Seeds of *M. truncatula* (A17) were produced as described in Chatelain *et al.* (2012). *Castanospermum australe* seeds were harvested during maturation and at shedding from trees growing in Pietermaritzburg (Kwazulu-Natal, South Africa) in 2009 and 2011. Within 48h after collection, they were air-freighted to Angers (France) where there were immediately processed as indicated. From the 2009 harvest, embryos and cotyledons from immature and mature seeds were separated. Cotyledon tissues were used for the critical water content determination or dried for 1 or 3 d over 75% relative humidity (RH) NaCl before being frozen in liquid nitrogen then stored at –80°C for RNA sequencing (Illumina) and proteomics. From the 2011 harvest, cotyledons and embryos were extracted from immature (green pods), mid-mature (yellow pods), and mature (brown pods) seeds, and frozen fresh in liquid nitrogen then stored at –80 °C. The 2011 harvest was used for analysis by 454 to improve the sequence assembly.

Desiccation sensitivity of mature *C. australe* cotyledons was determined on 3×5×3 mm cubes that were isolated from the inner part of the cotyledons. Cubes were dried for the indicated time intervals over a saturated salt solution at 75% RH, after which they were divided into two halves. One half was used for water content determination, and the other half for viability assessment following incubation for 24h in a 1% (w/v) tetrazolium solution (Sigma-Aldrich, France). Red colour was quantified by pixel intensity on the image using ImageJ software (http://rsb.info.nih.gov/ij/). Water content was determined gravimetrically by weighing the seeds before and after drying in an oven for 48h at 96 °C. Viability assays were performed on four independent drying experiments of 50–100 cubes.

Two *M. truncatula* mutants with *Tnt1* insertions in the *ABI3* gene (NF3185, hereafter referred to as *Mtabi3-1*; and NF6003, *Mtabi3-2*) were obtained from the Samuel Noble Foundation (Oklahoma, USA). *Tnt1* insertions in two mutants were verified by PCR (see Supplementary Table S1 available at *JXB* online for primers). Mutant and wild-type lines (R108) were multiplied in a growth chamber according to Chatelain *et al.* (2012), and lines were backcrossed once for the *Mtabi3-1* and twice for the *Mtabi3-2* mutants.

Desiccation tolerance was determined on seeds that were harvested at different time points during development. Two to five replicates of 30–50 seeds were rapidly dried to 0.09g H_2_O g DW^–1^ over an airflow of 43% RH, and rehydrated after 2 d on filter paper at 20 °C in the dark. Seeds were considered desiccation tolerant when they germinated, scored by the protrusion of the radicle through the seed coat. For ABA insensitivity assays, triplicates of 40–50 freshly harvested seeds just prior to pod abscission (0.8–1.0g H_2_O g DW^–1^) were imbibed on filter paper on a range of ABA concentrations (mixed isomers, Sigma, St Louis, MO, USA) at 20 °C. ABA was dissolved in methanol prior to dilution in water. Control seeds were imbibed in the MeOH concentration corresponding to the highest ABA concentration (0.5% MeOH). Germination was scored after 14 d. For proteomic analysis, *Mtabi3-1* and *Mtabi3-2* seeds were harvested at the point of abscission, when seeds were still viable. For reverse transcription–PCR (RT–PCR) analysis, seeds were harvested at 24 days after planting (DAP).

### Cloning of *MtABI3*

To obtain the full-length sequence for *MtABI3*, genomic DNA was extracted from leaf material of *M. truncatula* A17 using the Nucleospin Food kit (Macherey Nagel). An inverse PCR was performed on 5 μg of genomic DNA that was digested with *Eco*RI (25U per 50 μl final volume; Promega, Madison, WI, USA), and ligated using T4 DNA ligase (50U per 450 μl final volume; Fermentas, Vilnius, Lithuania). The full length was amplified on the ligated DNA using the primers indicated in Supplementary Table S1 at *JXB* online that were designed based on the *MtABI3* fragment available in the expresssed sequence tag (EST) database (TC97588, the DFCI *Medicago truncatula* Gene Index v8). The full-length genomic DNA fragment (3 458bp) was cloned into pJet1.2 (CloneJET kit, Thermo Scientific, Bremen, Germany) and sequenced (for primers see Supplementary Table S1).

### RNA extraction, and sequencing and assembly

For *M. truncatula* seeds, total RNA was extracted using the nucleospin RNAplant kit (Macherey Nagel, Düren, Germany), and 10 μg of total RNA from each sample were DNase treated (Turbo DNase, Ambion) and purified (RNeasy MinElute Cleanup kit, Qiagen) according to the manufacturer’s instructions. Total RNA was extracted with phenol from cotyledons or embryonic axes of *C. australe* as described by Bove *et al.* (2005). The quantity, purity, and integrity of RNA were checked using a NanoDrop ND-1000 UV-VIS spectrophotometer (NanoDrop Technologies) and a bioanalyzer (Experion, BioRad). From the 2009 RNA pool, a cDNA library was prepared, normalized, and sequenced by GenXPro GmbH (Frankfurt am Main, Germany) using Illumina technology (Genome Analyser-IIx). From the 2011 RNA pool, a cDNA library was prepared, normalized, and sequenced by Eurofins (Ebersberg, Germany) using the 454 GS FLX+ technology. Reads obtained from each sequencing were assembled *de novo* in two steps: first with MIRA 3.4.0 (Chevreux *et al.*, 2004) then with DNA Dragon (SequentiX, http://www.sequentix.de/software_dnadragon.php). The detailed procedure is described in Supplementary Fig. S1 at *JXB* online.

### Functional annotation and classification

Contig annotation to known sequences by sequence similarity was performed using two *M. truncatula* nucleic databases: MT3.5 from the International Medicago Genome Annotation Group (IMGAG) and MtGI11 from the Dana-Farber Cancer Institute (DFCI) Medicago Gene Index. Contigs that remained unannotated after these two analyses were blasted using Blast2GO (version 2.6.0) (Götz *et al.*, 2008) against protein databases including all plant species: Swissprot and non-redundant protein from NCBI. Next, classification of *C. australe* annotations in Gene Ontology (GO) was performed by Blast2GO. GO terms were retrieved from public databases and mapped to each contig, after which the most specific ones were selected by an annotation rule. The detailed annotation workflow is described in Supplementary Fig. S1 at *JXB* online.

### RT–PCR

A 2 μg aliquot of *M. truncatula* wild type and *abi3-1* and *abi3-2* RNA was reverse transcribed according to the manufacturers’ instructions (Thermo Scientific). The resulting cDNAs were diluted 1:3. Primer sequences and annealing temperatures are provided in Supplementary Table S1 at *JXB* online. PCR was performed with DreamTaq (Fermentas) according to the manufacturer’s instructions.

### Protein extraction and 2D gel electrophoresis

Total soluble proteins were extracted in triplicate from 25 seeds of *M. truncatula* (A17, R108, or *Mtabi3-1* and *Mtabi3-2*) and 400mg of cotyledons of mature *C. australe* from a minimum of three seeds for each replicate (2009 harvests) according to Boudet *et al.* (2006), and the heat-stable proteins were recovered according to Chatelain *et al.* (2012). After centrifugation at 20 000 *g* at 4 °C, the pellet was successively washed with 100 μl of 80% acetone, 100% acetone, 80% ethanol, and 100% ethanol then resuspended in 300 μl of rehydration buffer for 36h according to Boudet *et al.* (2006). Protein concentration was assayed according to Bradford (1976). Heat-stable protein fractions of *M. truncatula* and *C. australe* (150 μg), as well as a 1:1 mix of both protein fractions (300 μg), were rehydrated and separated on 24cm immobilized non-linear pH 3–10 gradient strips (Bio-Rad, Hercules, CA, USA). Isoelectric focusing was performed at 20 °C, for 3h at 250V, then 4h at 6kV, followed by a gradual increase to 27 kVh at 6kV h^–1^ and to 40 kVh at 8kV h^–1^ in a Bio-Rad Protean isoelectric focusing cell. Size separation of proteins was performed on vertical polyacrylamide gels [12% (w/v) acrylamide] in a Ettan Daltsix Electrophoresis system (Amersham Biosciences, Orsay, France) according to Boudet *et al.* (2006) using a running buffer containing 15.6mM TRIS (pH 8.3), 120mM glycine, and 0.1% (w/v) SDS. Gels were stained with 0.08% (w/v) Brillant Blue G-Colloidal for 24h, and destained briefly in 5% (v/v) acetic acid and 25% (v/v) methanol, then in 25% (v/v) methanol for 8h. Stained gels were scanned at 63.5×63.5 resolution using a GS 800 scanner (Bio-Rad). At least three digitalized gels from three independent experiments (extraction, focalization, and migration) were analysed using the PDQuest 7.2.0 software (Bio-Rad). Spot intensities were normalized using the total quantity in valid spot method. A paired *t*-test was performed to analyse differences in intensity between *C. australe* and *M. truncatula* LEA proteins and between wild-type (R108) and *Mtabi3-1*/*Mtabi3-2* seeds.

### Mass spectrometry and protein identification

Spots of interest were excised from the 2D gels and subjected to in-gel tryptic digestion according to Chatelain *et al.* (2012). Tryptic fragments were analysed by LC-ESI-MS/MS (liquid chromatography-electrospray ionization-tandem mass spectrometry) spectroscopy using a nanoscale HPLC (Famos-Switchos-Ultimate system, LC Packings, Dionex, San Francisco, CA, USA) coupled to a hybrid quadrupole orthogonal acceleration time-of-flight mass spectrometer (Q-TOF Global, Micromass-Waters, Manchester, UK) as described in Boudet *et al.* (2006). Mass data were analysed with the Protein Lynx Global Server software (Micromass-Waters). Protein identification was performed by comparing the data with the UniProt sequence databank (date of release: August 2010) or with the TIGR Medicago EST databank (date of release: April 2010). For the *M. truncatula* heat-stable proteome, spots linked to LEA polypeptides were identified according to the reference gel published by Chatelain *et al.* (2012).

### Data submission

Raw sequence data from this article can be found in the Sequence Reads Archive database (NCBI) under BioProject PRJNA193308. The data on the ectopic expression of *MtABI3* in hairy roots discussed in this publication have been deposited in NCBI’s Gene Expression Omnibus (Edgar *et al.*, 2002) and are accessible through GEO Series accession number GSE44291 (http://www.ncbi.nlm.nih.gov/geo/query/acc.cgi?acc=GSE44291).

## Results

### Physiological description of *C. australe* seeds

**Fig. 1.**
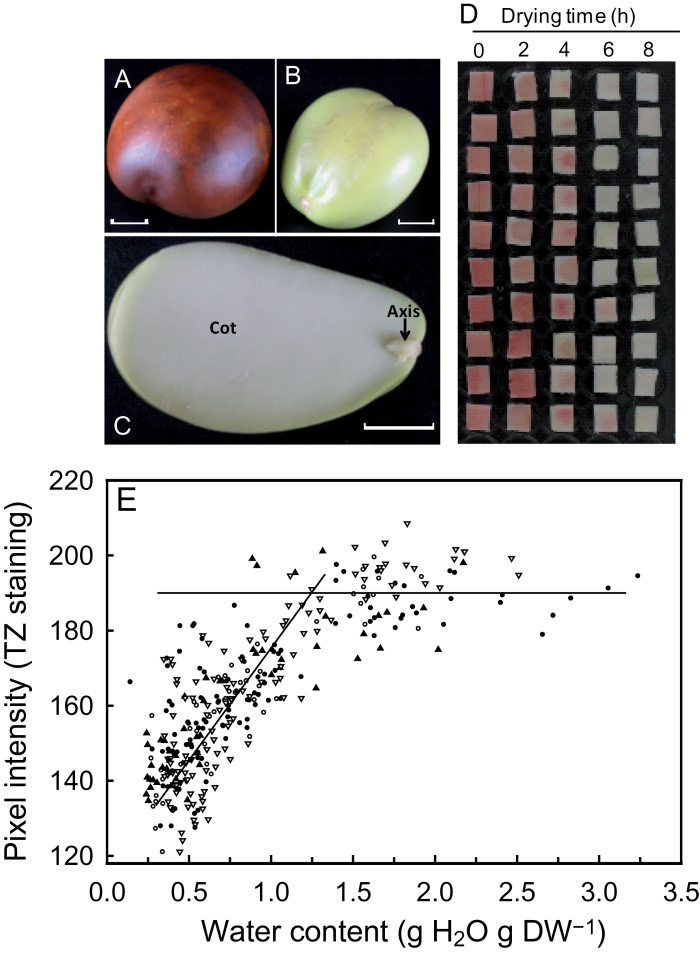
Determination of the critical water content of mature *Castanospermum australe* cotyledons. *Castanospermum australe* seed after shedding with (A) and without a seed coat (B). (C) Embryonic axis and cotyledons of mature seeds. The scale bar represents 1cm. (D) Tetrazolium staining (red indicating living tissues) of cubes (50mm^3^) taken from the core of mature cotyledons that were first dried at 44% RH for the indicated time. (E) The relationship between water content during drying and pixel intensity of the tetrazolium (TZ) staining. Data for four independent experiments (represented by different symbols) are shown.

On receipt, fresh weight and water content of mature, shed *C. australe* seeds were 45.9±14.9g per seed and 1.94±0.41g H_2_O g DW^–1^, respectively. The embryo is composed of two prominent cotyledons and a comparatively small axis, and is surrounded by a thin brown testa (~250 μm thickness) (Fig. 1A–C). When planted, fresh seeds germinated at 100% and produced healthy seedlings. The critical water content corresponding to the onset of loss of cell viability during rapid drying was determined on cotyledons using tetrazolium staining. During drying, the loss of viability as a function of water content showed the typical pattern found in other recalcitrant seeds (Fig. 1D, E). The intensity of the staining remained high and constant until a water content of ~1.5g H_2_O g DW ^–1^ was reached, after which the intensity decreased progressively with further drying (Fig. 1E). The critical water content, here defined as the water content corresponding to the break point for which cotyledon tissues begin to lose staining intensity, was estimated at 1.2g H_2_O g DW^–1^. Tissues were completely lacking red staining when dried below 0.5g H_2_O g DW^–1^ (Fig. 1D), indicating total loss of viability. This value is consistent with that reported by Han *et al.* (1997) on isolated axes of *C. australe* during rapid drying using electrolyte conductivity as an indication of membrane damage. In contrast, complete drying and rehydration of 10h imbibed *M. truncatula* cotyledons in tetrazolium solution rendered the tissues red, indicating that viability was maintained (data not shown). For the proteomic study, the focus was on cotyledons as a model for desiccation-sensitive tissues, due to their high critical water content. Cotyledons are large and surround the axis, thereby possibly slowing the rate of dehydration of the latter. In *M. truncatula*, few differences were found between LEA abundance and composition of the axes and cotyledons, except for EM6 (Chatelain *et al.*, 2012).

### Sequencing of the *C. australe* seed transcriptome and identification of LEA contigs

To enable comparative analysis on a molecular level between the recalcitrant *C. australe* and its orthodox counterpart *M. truncatula*, sequence information on transcripts present in seeds was obtained from a range of tissues to capture the maximum variation of the transcriptome at harvest: intact isolated axis, cotyledons from three developmental stages, and partially dried yet alive cotyledon tissues (Supplementary Fig. S1 at *JXB* online). Using Illumina and 454 technologies, sequencing of the normalized cDNA libraries resulted, respectively, in 7 784 004 paired reads of 76bp and 626 225 reads with an average length of 376bp (Table 1). The assembly resulted in 48 334 contigs varying between 200bp and 14 334bp long with an average length of 773bp (Table 1; Supplementary Fig. S1 at *JXB* online). A total of 35 050 contigs (72.5%) were annotated, of which 91% were provided by the IMGAG 3.5 *M. truncatula* database and the MtGI11 version of the Medicago EST database. An additional 740 and 2558 of the remaining contigs were identified, respectively, using Swissprot and NR databases related to other plant species (Supplementary Fig. S1). Annotations were classified according to GO using the Plant-GO-slim version of Blast2GO (B2G). Enzyme classification (EC) numbers were retrieved with the additional functionalities of B2G linked to KEGG pathways. A total of 23 637 contigs (48.9%) were annotated with 98 615 GO terms and 8 962 EC numbers. The distribution of the main biological processes (BP, 45 568 annotations), molecular functions (MF, 35 110 annotations), and cellular components (CC, 17 937 annotations) is shown in Supplementary Fig. S2.

**Table 1.** *Contig features from 454 and Illumina RNAseq of* Castanospermum australe *seeds and annotation of the corresponding transcriptome*

	454	Illumina	454+Illumina
Total number or reads after sequencing	626 225	7 784 004	8 410 229
Total number of contigs	36 767	18 483	48 334
Average contig length	869	318	773
N50	1001	345	1020
Number of nucleotides in contigs	31 937 738	5 882 730	37 365 884
Total number of contigs annotated	28 391	16 018	35 050
Contigs annotated with MT3.5	19 885	11 987	25 615
Contigs annotated with MtGI11	4 847	3 207	6 138
Contigs annotated with Swissprot	1 470	414	739
Contigs annotated with NR (NCBI)	2 189	410	2 558

Using the annotated transcriptome of *C. australe*, the next step was to perform a comparative analysis between LEA sequences found in both legume species. In *C. australe*, contigs were found for 29 LEA genes that are identified in the *M. truncatula* genome (Supplementary Table S2 at *JXB* online). In mature seeds of *M. truncatula*, a proteome analysis led to the detection of polypeptides corresponding to 17 genes (Chatelain *et al.*, 2012). For 16 out of these 17 genes, at least one corresponding contig was detected in the *C. australe* transcriptome (Table 2). Amino acid sequence alignment displayed between 52% and >90% similarity between the two species. The dehydrin family members were most divergent, with similarity that ranged only from 52% to 77% (Table 2). Other families are much more conserved between the two species, such as the SMP and LEA_5 family (EM1 and EM6), showing 76–86% identity and 85–91% similarity with *M. truncatula*, respectively.

**Table 2.** *LEA transcripts identified in the* Castanospermum *australe seed transcriptome*

Family (PFAM)	Protein name	Blast database	*M. truncatula* ID	*C. australe* contigs^*a*^	E-value	Alignment length	Percentage identity	Percentage similarity	Spot on 2D gel
Dehydrin	DHN3	MtGI11	TC175037	Ca_11990	2.E-09	198	47.2	52.0	3, 4
	DHN-cognate	Mt3.5	Medtr3g117290	Ca_9276	2.E-10	120	53.3	65.0	5
	BudCar5	Mt3.5	Medtr7g086340	Ca_31427	5.E-06	119	62.2	77.7	1
LEA_5	EM6	Mt3.5	Medtr4g016960	Ca_14307	4.E-45	98	81.6	90.8	37
	EM1	MtGI11	AJ498523	Ca_2036	9.E-40	100	78.2	85	51
LEA_4	SBP65	Mt3.5	Medtr4g079690	Ca_7340	5.E-17	90	58.9	72.2	75
	PM10	MtGI11	TC174929	Ca_3035	4.E-43	274	59.9	74.8	ND
	PM18	MtGI11	TC183861	Ca_330	2.E-41	335	56.0	67.2	ND
	MP2	Mt3.5	Medtr1g061730	Ca_8462	3.E-31	226	57.5	67.1	9
	LEAm	Mt3.5	Medtr2g014040	Ca_7604	3.E-45	268	48.6	62.4	74
	CAPLEA.I	MtGI11	TC175990	Ca_8841	1.E-31	141	66.0	85.8	18,19
LEA_1	PM1	Mt3.5	Medtr7g093170	Ca_8304	3.E-16	91	64.8	75.8	ND
	D113.II	Mt3.5	Medtr7g093160	Ca_8304	5.E-17	91	65.2	76.1	ND
SMP	PM25	MtGI11	TC174777	Ca_6007	2.E-83	238	75.6	84.4	92
	D-34.I	MtGI11	Medtr1g072090	ND					ND
	D-34.II	MtGI11	TC183570	Ca_25629	6.E-42	128	86.7	93	ND
	D-34.III	Mt3.5	Medtr2g076230	Ca_23377	3.E-06	24	76.0	87.5	ND

ND, homologue not detected.

Contigs were translated to calculate the percentage identity and similarity.

^*a*^ *Castanospermum australe* contigs that were homologues to the 17 LEA gene products detected in *M. truncatula* seeds were identified based on the MtGI11 and Mt3.5 *Medicago* databases (E-value <e-06).

### Identification of the heat-stable proteome of *C. australe*

**Fig. 2.**
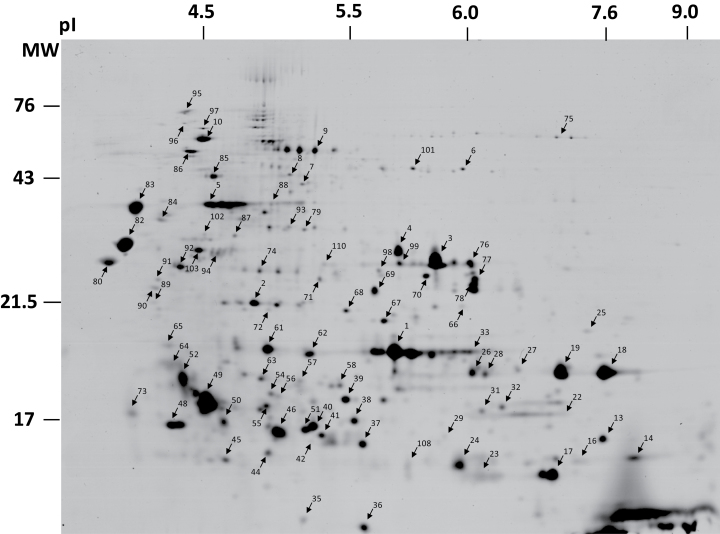
Reference map of the heat-stable proteome of mature *Castanospermum australe* cotyledons. A 150 μg aliquot of the heat-stable proteins was separated by 2D SDS–PAGE using 24cm non-linear immobilized pH gradient strips (3–10). pI and molecular mass (MW) (in kDa) are indicated. Numbers indicate the polypeptides that were sequenced (see Table 2; and Supplementary Table S3 at *JXB* online).

Identification of polypeptides corresponding to the 16 LEA transcripts that were detected in the *C. australe* transcriptome was carried out by separation of the heat-stable protein fraction by 2D gel electrophoresis (Fig. 2). This method has been successfully applied to characterize and quantify the entire LEA proteome of *M. truncatula* (Boudet *et al.*, 2006; Chatelain *et al.*, 2012). A total of 110 spots were sequenced using LC-ESI-MS/MS spectroscopy, out of which 82 spots were identified (Supplementary Table S3 at *JXB* online).

Polypeptides were detected for 10 of the 16 LEA genes identified from the *C. australe* sequence assembly (Table 2). These polypeptides include two highly abundant dehydrins [CaDHN3 (spot 3 and 4) and CaBudCar5 (spot 1)] and CaCAPLEA-1 (spot 18 and 19). Other less abundant LEA polypeptides include one more dehydrin (CaDHN-cognate, spot 5), the two LEA_5 members [CaEM1 (spot 51) and CaEM6 (spot 37)], three LEA_4 members [CaSBP65 (spot 75), CaMP2 (spot 9), and CaLEAm (spot 74)], and CaPM25 (spot 92). For six LEA contigs, no polypeptides were identified in the *C. australe* proteome, despite the presence of their transcripts (Table 2). In *M. truncatula*, four of these LEA proteins are highly abundant in mature seeds and include two members of the LEA_5 family (CaPM10 and CaPM18) and both LEA_1 members (CaD113.I and CaPM1) (Chatelain *et al.*, 2012). The other two LEA proteins that were not identified are members of the SMP family (CaD34.II and III).

In addition to LEA polypeptides, other abundant polypeptides were detected in the heat-stable *C. australe* proteome. They were identified as three pathogenesis-related proteins (spots 26, 27, and 29) (Supplementary Table S3 at *JXB* online), five polypeptides corresponding to small heat shock proteins (spots 2, 56, 61, 62, and 93), and four polypeptides corresponding to superoxide dismutases (38, 54, 67, and 69). Furthermore, two desiccation-related polypeptides (spots 82 and 88) were detected with homology to Lb_13-62 and PCC13-62. These genes are up-regulated in the desiccation-tolerant resurrection plants *Craterostigma plantagineum* and *Lindernia brevidens* (Phillips *et al.*, 2008) and were also recently detected in floral nectar of the evergreen velvet bean (*Mucuna sempervirens* Hemsl) (Zha *et al.*, 2013).

### Comparative analysis of the LEA proteome between *C. australe* and *M. truncatula*

**Fig. 3.**
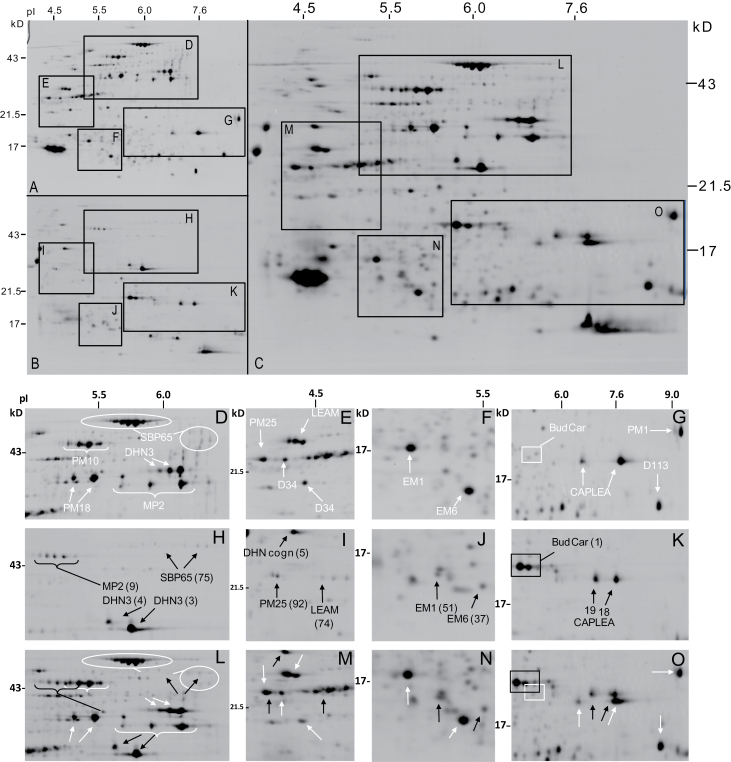
Comparative analysis of LEA polypeptides in the heat-stable proteome of cotyledons of *Castanospermum australe* and *Medicago truncatula* seeds. Reference map of the heat-stable proteome of *M. truncatula* (A) and *C. australe* seeds (B) and separation of a mixture of equal amounts of heat-stable proteins (150 μg) from both species (C). (D–O) Details of different regions of 2D gels of the proteome of *M. truncatula* (D–G), *C. australe* (H–K), and both species (L-O). The indicated spots refer to Supplementary Table S4 at *JXB* online.

The amount of heat-stable proteins relative to the total soluble protein fraction was lower for *C. australe* (20±1.8%) than for *M. truncatula* seeds (36%; Chatelain *et al.*, 2012). Equal amounts of the heat-stable protein fraction of *C. australe* or *M. truncatula* were separated by 2D gel electrophoresis, and 2D profiles were compared. For most of the LEA polypeptides, the exact position on the gel differed slightly between both species (Fig. 3A, B). This made it possible to combine the two extracts and separate them on the same gel, allowing for an accurate comparative quantification of the polypeptides from both species and avoiding the drawbacks associated with variations due to polypeptide migration and gel staining (Fig. 3C; Supplementary Table S4 at *JXB* online). In both species, the dehydrin DHN3 (Fig. 3D, H, L) and LEA_4 CAPLEA (Fig. 3G, K, O) were present with high spot intensity. For six LEA polypeptides, spot intensity was much lower in *C. australe* compared with *M. truncatula*; SBP65 and MP2 (Fig. 3D, H, L), PM25 and LEAm (Fig. 3E, I, M), and EM1 and EM6 (Fig. 3F, J, N) (Supplementary Table S4). The other two dehydrins, BudCar5 and DHN-cognate, are highly abundant in *C. australe* (Fig. 3I, K), whereas their homologues in *M. truncatula* are barely detectable (Fig. 3E, G).

**Fig. 4.**
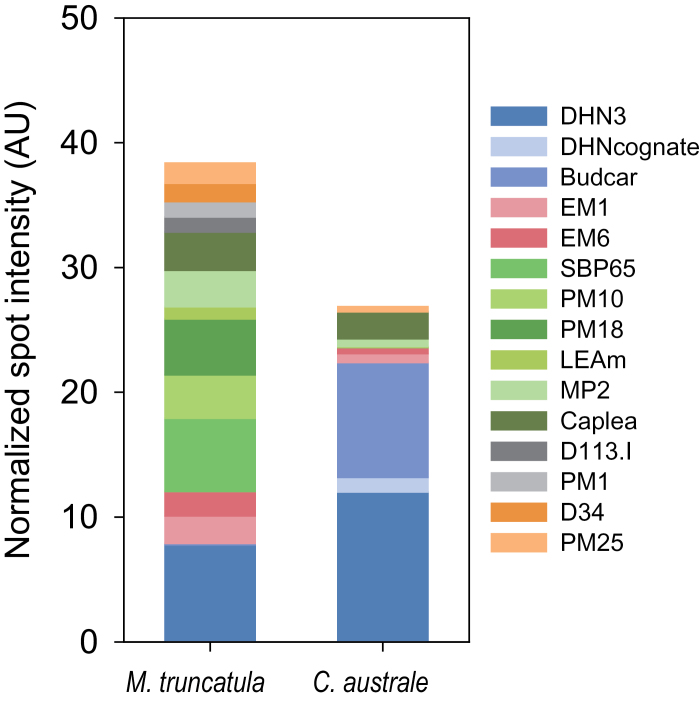
Relative abundance of the different LEA polypeptides identified in cotyledons of *Castanospermum australe* and *Medicago truncatula* seeds. Abundances were calculated based on the spot intensities of three replicates of the gels shown in Fig. 3.

A quantitative overview of the comparative analysis of the LEA proteome, based on relative spot intensity, is presented in Fig. 4. The LEA profile is strikingly different between the recalcitrant and orthodox seeds. In contrast to mature *M. truncatula* seeds, where dehydrins comprise 20% of the LEA proteome, this family represents 83% of the LEA proteome of *C*. *australe* cotyledons. Four LEA proteins (CaEM1, CaEM6, CaMP2, and CaPM25) were 4-fold less abundant in the recalcitrant seeds compared with the orthodox *M. truncatula*, whereas CaLEAm and CaSBP65 relative abundance was reduced >20-fold. In addition, six LEA proteins were not detected in cotyledons of *C. australe* (CaPM1, CaD113.I, two CaD34 members, CaPM10, and CaPM18). CAPLEA was present in comparable amounts in both species.

### Characterization of the LEA proteome of the desiccation-sensitive *Mtabi3* mutant seeds and comparison with *C. australe*

**Fig. 5.**
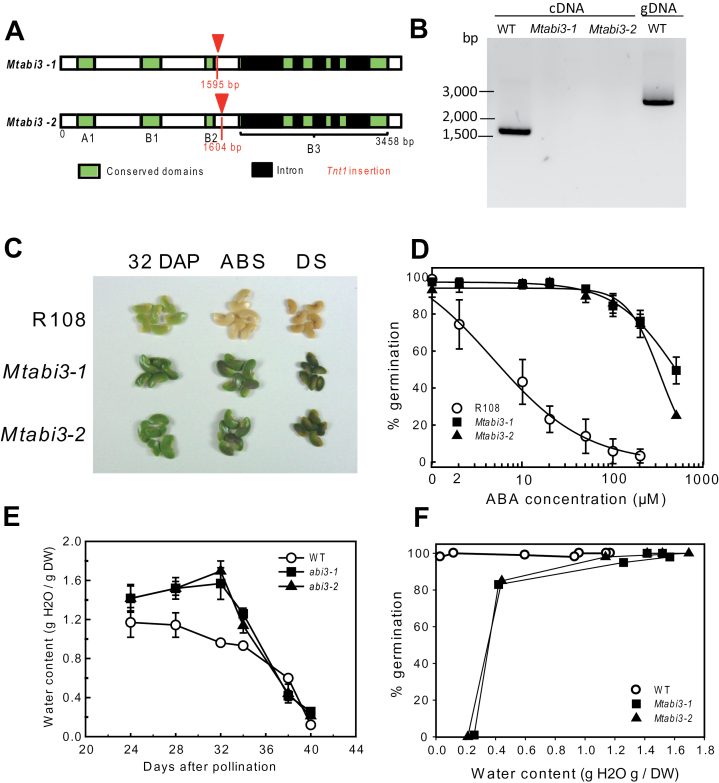
Characterization of *abscisic acid insensitive3* (*Mtabi3*) mutants of *Medicago truncatula*. (A) Gene structure, and position of the A and B domains and *Tnt1* insertions within the *MtABI3* gene. (B) Validation of the absence of ABI3 transcripts in the *Mtabi3-1* and *Mtabi3-2* mutants. Using the same primer set, ABI3 was also amplified on genomic DNA. The increased size corresponds to the additional introns. (C) Seed colour phenotype of *Mtabi3-1* and *Mtabi3-2* and corresponding wild-type seeds (R108) at three stages of maturation: 32 days after pollination (DAP), at pod abscission (ABS, 38 DAP), and in dry seed (DS). (D) ABA dose–response analysis during germination of seeds collected at pod abscission. Germination was scored as emergence of the radicle. Data are the average of three replicates of 40–50 seeds ±SE. (E) Changes in seed water content during development. Data are the average of three replicates of three seeds ±SE. (F) Germination of *Mtabi3* and wild-type seeds at different stages of development upon rehydration of 70–80 seeds. Data are significantly different when they differ by ≥18% (χ^2^ test, *P* < 0.05).

To investigate further a cause–effect relationship between the lack of these LEA proteins and desiccation sensitivity, the LEA proteome was examined in an orthodox seed that was rendered desiccation sensitive by knocking out *MtABI3* gene expression. First, two independent homozygous *Tnt1* insertion mutants (*Mtabi3-1* and *Mtabi3-2*) that were backcrossed once or twice, respectively, were obtained (Fig. 5A). The *Tnt1* insertions were located at 1 595bp and 1 605bp from the start codon, respectively, just after the B2 domain (Fig. 5A). RT–PCR analysis confirmed the absence of transcripts in the two *Mtabi3* mutants (Fig. 5B). The resulting freshly harvested seeds were used for a physiological characterization. Like in *abi3* mutants of *Arabidopsis* (Ooms *et al.*, 1993), mature *Mtabi3* seeds retained their chlorophyll (Fig. 5C) and exhibited a strongly reduced sensitivity to ABA (Fig. 5D). During seed maturation between 24 and 32 DAP, the seed water content of *abi3* mutants remained at ~1.6g H_2_O g DW^–1^, whereas in developing wild-type seeds it decreased steadily from 1.2g H_2_O g DW^–1^ to 1.0g H_2_O g DW^–1^ (Fig. 5E). Thereafter, in both *abi3* mutants and the wild type, the water content decreased until 40 DAP. During the latter stages of drying, when pods were detached, seeds of both genotypes exhibited a similar rate of water loss. Desiccation tolerance of harvested seeds was determined as a function of their water content at different stages during maturation and after enforced drying (at 40 DAP) (Fig. 5F). In contrast to the wild type, the seed population of *Mtabi3* mutants started to lose their viability when the water content decreased below 1.0g H_2_O g DW^–1^ and decreased sharply below 0.5g H_2_O g DW^–1^ (Fig. 5F). At 0.2g H_2_O g DW^–1^, all *abi3* seeds were dead. In contrast to wild-type seeds, fully mature, dried seeds did not germinate, and tetrazolium tests showed no staining, indicating that viability was completely lost (data not shown).

**Fig. 6.**
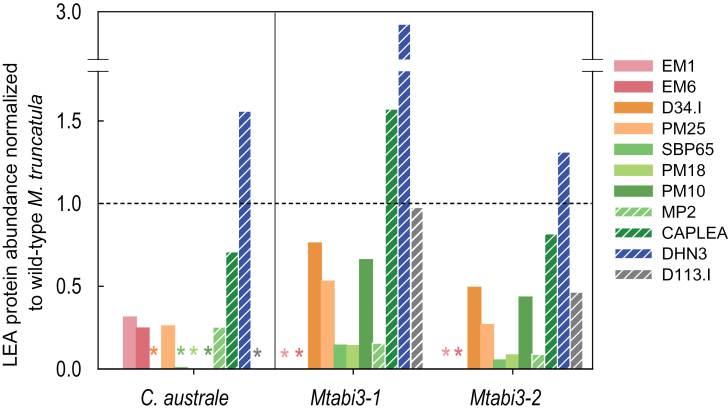
The LEA protein profile from desiccation-sensitive cotyledons of *Castanospermum australe* and seeds of *Mtabi3* mutants compared with desiccation-tolerant *Medicago truncatula* wild-type seeds. Abundance of LEA proteins in *C. australe* and *Mtabi3* (assessed as spot intensity, Supplementary Tables S4, S5 at *JXB* online) was normalized against their respective value obtained for wild-type *M. truncatula* seeds. A value of 1 corresponds to wild-type values (*C. australe*/A17 and *Mtabi3*/R108). Hatched bars correspond to non-seed-specific LEA proteins. Polypeptides whose abundance was not detected are indicated by asterisks.

Next, LEA polypeptide abundance was determined using 2D gel electrophoresis on three replicates of *Mtabi3-1*, *Mtabi3-2*, and wild-type seeds (R108 background) (Supplementary Table S5 at *JXB* online). To be able to compare LEA profiles among *C. australe* and *Mtabi3* genotypes, polypeptide abundance was expressed as the relative difference from *M. truncatula* wild type (A17 for the comparison with *C. australe*, and R108 for the comparison with *Mtabi3* mutants) (Fig. 6). The intensity of MtPM1 was highly variable amongst the samples, irrespective of the *M. truncatula* genotypes (Supplementary Table S5). This might be due to the very basic nature of this protein in the R108 genotype, placing it at the border of the gel where resolution is poor. Likewise, the intensity of MtLEAm and D34.II could not be determined correctly (Fig. 3; Supplementary Table S5). To avoid incorrect interpretation of these data, they were omitted from further analysis. Overall, the abundance of the LEA proteome of the *Mtabi3* mutants compared with wild-type seeds resembled that of desiccation-sensitive *C. australe* cotyledons when compared with *M. truncatula* (Fig. 6). As in *C. australe*, the abundance of nine LEA polypeptides from several families was decreased in the *Mtabi3* mutants, namely LEA_5 (MtEM1 and MtEM6), SMP (MtPM25 and MtD34), LEA_4 (MtSBP65, MtPM18, MtPM10, and MtMP2), and LEA_1 (D113.I). The dehydrin MtDHN3 was more abundant in seeds of *Mtabi3* mutants than in wild-type seeds, which further underscored the similarity with *C. australe*. The relative amount of MtCAPLEA was slightly lower in seeds of the *Mtabi3-2* mutant compared with the wild-type seeds, whereas its amount was higher in the *Mtabi3-1* mutant (Fig. 6; Supplementary Table S5).

### ABI3 regulation of identified LEA proteins in relation to desiccation tolerance

The reduction of LEA polypeptides in *Mtabi3* mutants raises the question of whether the 12 reduced or absent LEA proteins in *C. australe* are regulated by ABI3 at the gene level. In *abi3* seeds of *Arabidopsis*, transcript levels of all LEA genes that are affected in the desiccation-sensitive tissues (group A) were decreased (Table 3; Bies-Etheve *et al.*, 2008). This was not the case for the LEA proteins that were not affected or that were more abundant in these tissues (group B). Transcript levels of the homologues of *DHN-cognate* and *CAPLEA1* were even increased in the *abi3* mutants. In addition, to investigate whether MtABI3 regulates LEA targets, advantage was taken of a recent transcriptome study on the effect of overexpressing *MtABI3* in the hairy roots of *M. truncatula* (GeOmnibus GS GSE44291). The advantage of this ectopic expression model is that it avoids the interfering effects of other B3 domain transcription factors such as FUS3 and LEC2 with ABI3 in seeds (Mönke *et al.*, 2012). Transcript levels of 9 out of 11 genes coding for group A LEA proteins were up-regulated by *MtABI3* (Table 3). Moreover, *in silico* promoter analysis of 8 out of 10 LEA genes for which promoter sequences could be retrieved indicated that seven LEA promoters from group A contain both RY (CATGCA) and ABRE (ACGTG(G/T)C) *cis*-regulatory elements. The RY element is known to be bound by ABI3, while ABRE motifs are implicated in the binding of bZIP-TFs, known to interact with ABI3 (Busk *et al.*, 1997; Hattori *et al.*, 2002; Guerriero *et al.*, 2009). A study on the identification of the ABI3 regulon in *Arabidopsis* confirmed three LEA genes as direct targets by transient promoter activation assay or ChIP-chip analysis (Table 3). The other LEA proteins were identified as being ABI3-responsive gene products in *35S::ABI3-GR* seedlings (Mönke *et al.*, 2012). An analysis of the other four LEA proteins that were abundant in *C. australe* cotyledons (group B, Table 3) demonstrated that overexpressing *MtABI3* in *M. truncatula* roots induced *DHN3* trancripts and slightly activated *BudCar5*, although transcript levels were found to increase in *abi3* mutants (Table 3). No effect was found on transcript levels of *CAPLEA-1*, and *DHN-cognate* transcripts even decreased significantly. No RY element was retrieved in promoter analysis of *BudCar5* and *DHN-cognate* coding genes, and neither gene was part of the ABI3 regulon identified by Monke *et al.* (2012).

**Table 3.** *Evidence for ABI3-dependent regulation of LEA homologues for which protein abundance is reduced or absent in desiccation-sensitive tissues (*C. australe *and* Mtabi3*) (group A) or unaffected or increased (group B)*

Protein name	LEA group	*Medicago truncatula*				*Cis*-elements^*b*^	*Arabidopsis thaliana*		
		Sequence ID	Nimblegen probe	35S::ABI3/ control^*a*^	*P*-value		AGI	Transcript level in *abi3* seeds versus WT^*c*^	ABI3 targets^*d*^
EM6	A	Medtr4g016960	Medtr_v1_022627	3.87	1.92E-06	2 RY, 2 ABRE	AT2G40170	Down	–
EM1	A	AJ498523	Medtr_v1_072582	2.04	5.53E-02	ND	AT3G51810	Down	T, P
SBP65	A	Medtr4g079690	Medtr_v1_083614	4.18	3.07E-03	2 RY, 1 ABRE	AT2G42560	Down	T, P
PM10	A	Medtr8g134020	Not present on slide			ND	AT5G44310	NA	T, P
LEAm	A	Medtr2g014040	Medtr_v1_009629	2.98	2.34E-03	1 RY, 2 ABRE	AT5G44310	NA	T, P
MP2	A	Medtr1g061730	Medtr_v1_005821	3.87	1.51E-05	0 RY, 2 ABRE	AT2G36640	Down	T, P
PM18	A	TC183861	Medtr_v1_076240	–0.02	8.65E-01	ND	AT2G36640	Down	T, P
PM1	A	Medtr7g093170	Medtr_v1_045826	4.59	7,89E-07	0 RY, 1 ABRE	AT5G06760	NA	C
D113.II	A	Medtr7g093160	Medtr_v1_045826	4.59	7.89E-07	2 RY, 1 ABRE	AT5G06760	NA	C
PM25	A	TC174777	Medtr_v1_082683	3.29	1.32E-02	ND	AT3G22490	Down	T, P, A
D-34.I	A	Medtr1g072090	Medtr_v1_006041	2.21	4.07E-02	5 RY, 0 ABRE	AT3G22490	Down	T, P, A
D-34.III	A	Medtr2g076230	Medtr_v1_012326	2.32	4.43E-02	0 RY, 0 ABRE	AT3G22490	Down	T, P, A
DHN3	B	TC175037	Medtr_v1_066754	3.32	1.10E-05	ND	ND	ND	ND
DHN-cognate	B	Medtr3g117290	Medtr_v1_020587	–1.47	4.91E-04	0 RY, 1 ABRE	AT1G76180	Up	–
BudCar5	B	Medtr7g086340	Medtr_v1_045277	1.57	3.70E-02	0 RY, 0 ABRE	ND	ND	ND
CAPLEA-1	B	TC175990	Medtr_v1_085905	1.06	6.96E-02	ND	AT1G52690	Up	–

^*a*^ Log ratio of transcript levels (and corresponding *P*-values) in hairy roots overexpressing *MtABI3* compared with control (empty plasmid), determined by trancriptome analysis using Nimblegen slides (GeOmnibus GSE44291).

^*b*^ The number of RY (CATGCA) and ABRE (ACGTG(G/T)C) *cis*-regulatory motifs known to bind ABI3 was revealed by analysing the 2kb promoter sequence of the *M. truncatula* genes.

^*c*^ Relative level of transcripts in mature *abi3* seeds of *Arabidopsis* compared with the wild type. Data are extracted from Bies-Etheve *et al.* (2008).

^*d*^ Identification of ABI3-responsive gene products in *35S::ABI3-GR* seedlings by array-based transcriptome analysis (T) or qRT-PCR (P) and confirmation as direct targets by transient promoter activation assay (A) or ChIP-chip analysis (C). Data are extracted from Mönke *et al.* (2012).

ND, not detected; NA, not analysed; WT, wild type.

## Discussion

The aim of this study was to compare the seed LEA proteome of two legume species exhibiting orthodox and recalcitrant storage behaviour to gain further insights into the panoply of these protective proteins necessary for desiccation tolerance. This work shows that *C. australe* and *M. truncatula*, both from the *Papilionaceae* subfamily of Fabaceae, are phylogenetically close enough to allow for a detailed sequence comparison of LEA accumulation in relation to desiccation tolerance. Assembly of a normalized sequencing library identified contigs with high similarity for 16 of the 17 *M. truncatula* LEA genes (Table 2) for which protein accumulation was shown in *M. truncatula* (Chatelain *et al.*, 2012). This comparison was further extended to a desiccation-sensitive *Mtabi3* mutant of *M. truncatula* that was obtained and characterized.

It is believed that this is the first report of full coverage of the identification of the LEA genes and their products (the ‘LEAome’) in cotyledons (the predominant tissue in this species) of a recalcitrant seed. To date, studies have been constrained to dehydrins using an antibody against the consensus sequence KIKEKLPG (Berjak and Pammenter, 2008). The comparison with *M. truncatula* revealed that 12 out of 16 LEA proteins are less abundant or not detected in the recalcitrant *C. australe* seed proteome (Figs 4, 6). *In silico* gene expression analysis of *M. truncatula* transcriptomes demonstrated that all but one (*MtMP2*) of these 12 genes are specifically expressed in seed tissues (Chatelain *et al.*, 2012). Further LEA proteome analysis of the *Mtabi3* mutants revealed that accumulation of the homologues of these LEA proteins was affected in these desiccation-sensitive seeds. Several of them (MtSBP65, MtPM25, MtEM6, MtPM18, and MtMP2) correlated with the re-induction of desiccation tolerance in germinated radicles of *M. truncatula* seeds (Boudet *et al.*, 2006). Figure 5E and F shows that seeds of *Mtabi3* mutants can survive drying to 0.4g H_2_O g DW^–1^ and can be considered drought tolerant. They lose their viability after they are shed from the mother plant. In *C. australe*, tissues did not survive drying down to 0.5g H_2_O g DW^–1^. Collectively, these data suggest that these particular LEA proteins are needed once bulk water is removed. For most of them, their role in the dry state is not yet elucidated. *In vitro* studies of EM6, PM25 (Boudet *et al.*, 2006; Gilles *et al.*, 2007; Boucher *et al.*, 2010), and LEAm (Tolleter *et al.*, 2007) demonstrated multifunctional protective capacities with different efficiencies. These include membrane (LEAm) and enzyme protection (LEAm, EM6, PM25), anti-aggregation against thermo-mechanical stress (EM6 and PM25), and water binding (EM6 and PM25).

This work offers a new model to study the regulatory and mechanistic pathways implicated in desiccation tolerance through comparative analysis of desiccation-sensitive cotyledon tissues from recalcitrant seeds and their orthodox counterparts. The proteome comparison with *Mtabi3* seeds suggests that comparable pathways leading to LEA accumulation are affected in both desiccation-sensitive orthodox and recalcitrant cotyledon tissues (Fig. 6). Consistent with this observation, homologous LEA genes in *Arabidopsis* and *M. truncatula* are ABI3 responsive (Table 3). It is not known whether the reduced LEA polypeptides in *C. australe* cotyledons are linked to reduced *CaABI3* activity or defective upstream or downstream signalling pathways. Interestingly, a *CaABI3* contig was detected in the RNA assembly, but its temporal and spatial expression, as well as its efficiency need to be assessed. In developing orthodox seeds, the RY *cis*-elements are elements that are crucial for transactivation through ABI3/VP1-like B3-domain proteins, whereas conserved ABA-responsive elements (ABREs; PyACGTGG/TC) mediate ABI3-related ABA signalling in conjunction with other transcription factors, such as ABI5 (Busk *et al.*, 1997; Hattori *et al.*, 2002; Guerrriero *et al.*, 2009). Most of the LEA promoters in *Medicago* for which protein abundance was decreased or absent in *Castanospermum* were found to contain both RY and ABRE elements (Table 3). The LEA genes for which protein abundance was not affected or even increased in *C. australe* compared with *M. truncatula* do not seem to be regulated by ABI3 (Table 3). Transcript levels of DHN-cognate even increased in the *abi3* mutants of *Arabidopsis* and decreased in transgenic roots when overexpressing *MtABI3* (Table 3). Whether this gene is negatively regulated by ABI3 is unknown. Taken together, these results strengthen the idea that only LEA proteins positively regulated by ABI3 are reduced in *C. australe* cotyledons. However, it is likely that additional regulatory pathways intervene in the accumulation of these desiccation-tolerant associated proteins because in both *C. australe* and the *Mtabi3* mutants, a number of LEA proteins were not absent but their levels were partially reduced. Other transcription factors that regulate LEA gene expression are *ABI4* and *ABI5* (Bies-Etheve *et al.*, 2008; Reeves *et al.*, 2011). ABI3 interacts with ABI5 to regulate expression of downstream genes, whereas ABI4 controls the induction of ABI5 (Bossi *et al.*, 2009; Cutler *et al.*, 2010). However, in *Arabidopsis*, mature seeds of *abi4* and *abi5* mutants are desiccation tolerant. In addition, loss-of-function *lec1* mutants of *Arabidopsis* produce seeds that lose their viability during desiccation or during the ﬁrst few weeks after harvest (Meinke, 1992). However, an analysis of direct targets of LEC1 did not reveal any LEA genes (Bäumlein and Junker, 2012; Wang and Perry, 2013). Considering that homologues of *ABI3*, *LEC1*, *ABI5*, and *FUS3* were detected in the *C. australe* RNAseq assembly, the role of these transcription factors in seed development warrants further investigation, particularly in relation to its recalcitrant behaviour.

Sequencing of the *C. australe* transcriptome was performed by high-throughput sequencing 454 and Illumina technologies on a normalized library. Library normalization improves the proportion of low abundant sequences and maximizes transcriptome coverage (Zhulidov *et al.*, 2004). Both technologies have been extensively used in the past few years to sequence transcriptomes of non-model species without a reference genome (reviewed in Schliesky *et al.*, 2012). Table 1 confirms that hybrid *de novo* assembly combining both sequencing technologies improves transcriptome coverage, as suggested by Wall *et al.* (2009) and Garg *et al.* (2011). More than 72% of the 48 334 contigs could be annotated by this approach, and 91% of this annotation is provided by *M. truncatula*-specific databases. This approach also enabled the discovery of almost all LEA transcripts for comparison with *M. truncatula*. However, a quantitative transcriptome analysis will be needed to reveal to what extent LEA polypeptide abundance is regulated at the transcriptional and/or post-transcriptional level in *C. australe*. Furthermore, there are many other molecular protective mechanisms that could be missing in this recalcitrant species such as antioxidant defences, non-reducing sugars, heat shock proteins, and/or induction of cell wall modifications (reviewed in Berjak and Pammenter, 2008; Leprince and Buitink, 2010). The sequence assembly from the normalized library will enable the construction of microarrays to investigate further molecular aspects of desiccation sensitivity in recalcitrant seeds.

A striking observation was the highly increased amount of dehydrins in the recalcitrant seeds compared with *M. truncatula* (Fig. 6). Two of them (BudCar5 and DHN3) have also been identified in desiccation-sensitive seedlings of *M. truncatula* submitted to osmotic stress (Boudet *et al.*, 2006). Furthermore, *in silico* analysis using the *Medicago* gene atlas shows that these dehydrins are expressed in many different organs other than seeds in stressful conditions (Benedito *et al.*, 2008; Chatelain *et al.*, 2012). One can speculate on the functional role for such proteins in recalcitrant seeds (Berjak and Pammenter, 2008). Most recalcitrant seeds are spheroid, with large cotyledons surrounding the axis. The synthesis of dehydrins in cotyledons can protect the axis from the dehydration stress that they will undergo after shedding. Furthermore, due to their size, dehydration is likely to be slow and thus a requirement for protection against only mild water deficit stress should be sufficient for maintenance of seed viability as a whole in seeds shed into their natural environmental habitat. Dehydrins are known to increase tolerance to osmotic stress, demonstrated by the overexpression of dehydrin *Rab17* and *Rab28* in *A. thaliana* plants and maize plants, respectively (Figueras *et al.*, 2004; Amara *et al.*, 2013).

In conclusion, the comparative analysis of the LEA proteome proﬁles of two unrelated desiccation-sensitive tissues (cotyledons of *C. australe* and seeds of *Mtabi3*) with the orthodox *M. truncatula* indicates that the developmental programme leading to desiccation tolerance involves the synthesis of a variety of seed-specific LEA proteins that have been poorly characterized so far and partially involves ABI3. This developmental programme is intertwined with the synthesis of additional LEA proteins such as dehydrins as an apparent need to retain some tolerance against mild osmotic stress during maturation.

## Supplementary data

Supplementary data are available at *JXB* online.

Figure S1. Sequencing, assembly, and annotation workflow of the *C. australe* seed transcriptome.

Figure S2. GO annotation of the sequence assembly of the transcriptome of *C. australe* seeds.

Table S1. Primer sequences used for PCR.

Table S2. Overview of contigs from the *C. australe* transcriptome matching LEA-coding genes of *M. truncatula*.

Table S3. Summary of identified spots from the reference gel of the heat-soluble protein fraction of *C. australe* cotyledons.

Table S4. Normalized intensity of polypeptides of the heat-stable proteome of *M. truncatula* and *C. australe* seeds.

Table S5. Normalized intensity of polypeptides of the heat-stable proteome of *M. truncatula* R108 (wild type), *Mtabi3-1*, and *Mtabi3-2* seeds.

Supplementary Data

## References

[CIT0001] Amara I Capellades M Ludevid MD Pagès M Goday A 2013 Enhanced water stress tolerance of transgenic maize plants over-expressing LEA Rab28 gene. Journal of Plant Physiology 170, 864––873 23384757 10.1016/j.jplph.2013.01.004

[CIT0002] Amara I Odena A Oliveira E Moreno A Masmoudi K Pages M Goday A 2012 Insights into maize LEA proteins: from proteomics to functional approaches. Plant and Cell Physiology 53, 321––329 10.1093/pcp/pcr183 22199372

[CIT0003] Battaglia M Olvera-Carrillo Y Garciarrubio A Campos F Covarrubias AA 2008 The enigmatic LEA proteins and other hydrophilins. Plant Physiology 148, 6––24 18772351 10.1104/pp.108.120725 PMC2528095

[CIT0004] Bäumlein H Junker A 2012 Multifunctionality of the LEC1 transcription factor during plant development. Plant Signaling Behavior 7, 1718––1720 23073004 10.4161/psb.22365 PMC3578918

[CIT0005] Benedito VA Torres-Jerez I Murray JD 2008 A gene expression atlas of the model legume *Medicago truncatula* . The Plant Journal 55, 504––513 18410479 10.1111/j.1365-313X.2008.03519.x

[CIT0006] Berjak P Pammenter NW 2008 From *Avicennia* to *Zizania*: seed recalcitrance in perspective. Annals of Botany 101, 213––228 17704237 10.1093/aob/mcm168 PMC2711015

[CIT0007] Bies-Etheve N Gaubier-Comella P Debures A Lasserre E Jobet E Raynal M Cooke R Delseny M 2008 Inventory, evolution and expression profiling diversity of the LEA (late embryogenesis abundant) protein gene family in *Arabidopsis thaliana* . Plant Molecular Biology 67, 107––124 18265943 10.1007/s11103-008-9304-x

[CIT0008] Bossi F Cordoba E Dupré P Mendoza MS Román CS León P 2009 The Arabidopsis ABA-INSENSITIVE (ABI) 4 factor acts as a central transcription activator of the expression of its own gene, and for the induction of ABI5 and SBE2.2 genes during sugar signaling. The Plant Journal 59, 359––374 19392689 10.1111/j.1365-313X.2009.03877.x

[CIT0009] Boucher V Buitink J Lin X Boudet J Hoekstra FA Hundertmark M Renard D Leprince O 2010 MtPM25 is an atypical hydrophobic late embryogenesis-abundant protein that dissociates cold and desiccation-aggregated proteins. Plant, Cell and Environment 33, 418––430 10.1111/j.1365-3040.2009.02093.x 20002332

[CIT0010] Boudet J Buitink J Hoekstra FA Rogniaux H Larré C Satour P Leprince O 2006 Comparative analysis of the heat stable proteome of radicles of *Medicago truncatula* seeds during germination identifies Late Embryogenesis Abundant proteins associated with desiccation tolerance. Plant Physiology 140, 1418––1436 16461389 10.1104/pp.105.074039 PMC1435805

[CIT0011] Bove J Lucas P Godin B Ogé L Jullien M Grappin P 2005 Gene expression analysis by cDNA-AFLP highlights a set of new signaling networks and translational control during seed dormancy breaking in *Nicotiana plumbaginifolia.* Plant Molecular Biology 57, 593––612 15821982 10.1007/s11103-005-0953-8

[CIT0012] Bradford MM 1976 A rapid and sensitive method for the quantitation of microgram quantities of protein utilizing the principle of protein–dye binding. Analytical Biochemistry 72, 248––254 942051 10.1016/0003-2697(76)90527-3

[CIT0013] Buitink J Leger JJ Guisle I 2006 Transcriptome profiling uncovers metabolic and regulatory processes occurring during the transition from desiccation-sensitive to desiccation-tolerant stages in *Medicago truncatula* seeds. The Plant Journal 47, 735––750 16923015 10.1111/j.1365-313X.2006.02822.x

[CIT0014] Busk PK Jensen AB Pages M 1997 Regulatory elements *in vivo* in the promoter of the abscisic acid responsive gene rab17 from maize. The Plant Journal 11, 1285––195 9225468 10.1046/j.1365-313x.1997.11061285.x

[CIT0015] Chatelain E Hundertmark M Leprince O le Gall S Satour P Deligny-Penninck S Rogniaux H Buitink J 2012 Temporal profiling of the heat stable proteome during the late maturation of *Medicago truncatula* seeds identifies a restricted subset of late embryogenesis abundant proteins associated with longevity. Plant, Cell and Environment 35, 1440––1455 10.1111/j.1365-3040.2012.02501.x 22380487

[CIT0016] Chevreux B Pfisterer T Drescher B Driesel AJ Müller WEG Wetter T Suhai S 2004 Using the miraEST assembler for reliable and automated mRNA transcript assembly and SNP detection in sequenced ESTs. Genome Research 14, 1147––1159 15140833 10.1101/gr.1917404 PMC419793

[CIT0017] Cutler SR Rodriguez PL Finkelstein RR Abrams SR 2010 Abscisic acid: emergence of a core signaling network. Annual Review of Plant Biology 61, 651––679 10.1146/annurev-arplant-042809-112122 20192755

[CIT0018] Doyle JJ 1995 DNA data in legume phylogeny: a progress report. In: Crisp MD Doyle JJ, eds. Advances in legume systematics part 7. Phylogeny. London: The Royal Botanic Gardens Kew, 11––30

[CIT0019] Edgar R Domrachev M Lash AE 2002 Gene Expression Omnibus: NCBI gene expression and hybridization array data repository. Nucleic Acids Research 30, 207––210 11752295 10.1093/nar/30.1.207 PMC99122

[CIT0020] Farrant JM Pammenter NW Berjak P Farnsworth EJ Vertucci CW 1996 Presence of dehydrin-like proteins and levels of abscisic acid in recalcitrant (desiccation sensitive) seeds may be related to habitat. Seed Science Research 6, 175––182

[CIT0021] Farnsworth E 2000 The ecology and physiology of viviparous and recalcitrant seeds. Annual Review of Ecology and Systematics 31, 107––138

[CIT0022] Figueras M Pujal J Saleh A Save R Pagès M Goday A 2004 Maize Rabl7 overexpression in Arabidopsis plants promotes osmotic stress tolerance. Annals of Applied Biology 144, 251––257

[CIT0023] Finch-Savage WE Pramanik SK Bewley JD 1994 The expression of dehydrin proteins in desiccation-sensitive (recalcitrant) seeds of temperate trees. Planta 193, 478––485

[CIT0024] Garg R Patel RK Jhanwar S Priya P Bhattacharjee A Yadav G Bhatia S Chattopadhyay D Tyagi AK Jain M 2011 Gene discovery and tissue-specific transcriptome analysis in chickpea with massively parallel pyrosequencing and web resource development. Plant Physiology 156, 1661––1678 21653784 10.1104/pp.111.178616 PMC3149962

[CIT0025] Gilles GJ Hines KM Manfre AJ Marcotte WR 2007 A predicted N-terminal helical domain of a Group 1 LEA protein is required for protection of enzyme activity from drying. Plant Physiology and Biochemistry 45, 389––399 17544288 10.1016/j.plaphy.2007.03.027

[CIT0026] Götz S García-Gómez JM Terol J Williams TD Nagaraj SH Nueda MJ Robles M Talón M Dopazo J Conesa A 2008 High-throughput functional annotation and data mining with the Blast2GO suite. Nucleic Acids Research 36, 3420––3435 18445632 10.1093/nar/gkn176 PMC2425479

[CIT0027] Guerriero G Martin N Golovko A Sundstrom JF Rask L Ezcurra I 2009 The RY/Sph element mediates transcriptional repression of maturation genes from late maturation to early seedling growth. New Phytologist 184, 552––565 19659659 10.1111/j.1469-8137.2009.02977.x

[CIT0028] Han B Berjak P Pammenter N Farrant J Kermode AR 1997 The recalcitrant plant species, *Castanospermum australe* and *Trichilia dregeana*, differ in their ability to produce dehydrin-related polypeptides during seed maturation and in response to ABA or water-deficit-related stresses. Journal of Experimental Botany 48, 1717––1726

[CIT0029] Hattori T Totsuka M Hobo T Kagaya Y Yamamoto-Toyoda A 2002 Experimentally determined sequence requirement of ACGT-containing abscisic acid response element. Plant and Cell Physiology 43, 136––140 11828032 10.1093/pcp/pcf014

[CIT0030] Hinniger C Caillet V Michoux F Ben Amor M Tanksley S Lin CW McCarthy J 2006 Isolation and characterization of cDNA encoding three dehydrins expressed during *Coffea canephora* (Robusta) grain development. Annals of Botany 97, 755––765 16504969 10.1093/aob/mcl032 PMC2803416

[CIT0031] Hundertmark M Buitink J Leprince O Hincha DK 2011 The reduction of seed-specific dehydrins reduces seed longevity in *Arabidopsis thaliana* . Seed Science Research 21, 165––173

[CIT0032] Hundertmark M Hincha DK 2008 LEA (Late Embryogenesis Abundant) proteins and their encoding genes in *Arabidopsis thaliana* . BMC Genomics 9, 118 18318901 10.1186/1471-2164-9-118 PMC2292704

[CIT0033] Illing N Denby KJ Collett H Shen A Farrant JM 2005 The signature of seeds in resurrection plants: a molecular and physiological comparison of desiccation tolerance in seeds and vegetative tissues. Integrative Comparative Biology 45, 771––787 21676829 10.1093/icb/45.5.771

[CIT0034] Ismail FA Nitsch LMC Wolters-Arts MMC Mariani C Derksen JWM 2010 Semi-viviparous embryo development and dehydrin expression in the mangrove *Rhizophora mucronata* Lam. Sexual Plant Reproduction 23, 95––103 20084524 10.1007/s00497-009-0127-y PMC2874033

[CIT0035] Kermode AR 1997 Approaches to elucidate the basis of desiccation-tolerance in seeds. Seed Science Research 7, 75––96

[CIT0036] Kotak S Vierling E Baumlein H Koskull-Doring P 2007 A novel transcriptional cascade regulating expression of heat stress proteins during seed development of *Arabidopsis* . The Plant Cell 19, 182––195 17220197 10.1105/tpc.106.048165 PMC1820961

[CIT0037] Lee AK Slovin JP Suh JK 2012 Dehydration intolerant seeds of Ardisia species accumulate storage and stress proteins during development. Horticulture, Environment, and Biotechnology 53, 530––538

[CIT0038] Leprince O Buitink J 2010 Desiccation tolerance: from genomics to the field. Plant Science 179, 554––564

[CIT0039] Li DZ Pritchard HW 2009 The science and economics of ex situ plant conservation. Trends in Plant Science 14, 614––621 19818672 10.1016/j.tplants.2009.09.005

[CIT0040] Manfre AJ LaHatte GA Climer CR Marcotte WR 2009 Seed dehydration and the establishment of desiccation tolerance during seed maturation is altered in the *Arabidopsis thaliana* mutant *atem6-1* . Plant and Cell Physiology 50, 243––253 19073649 10.1093/pcp/pcn185

[CIT0041] Meinke DW 1992 A homoeotic mutant of *Arabidopsis thaliana* with leafy cotyledons. Science 258, 1647––1650 17742538 10.1126/science.258.5088.1647

[CIT0042] Mönke G Seifert M Keilwagen J 2012 Toward the identification and regulation of the *Arabidopsis thaliana* ABI3 regulon. Nucleic Acids Research 40, 8240––8254 22730287 10.1093/nar/gks594 PMC3458547

[CIT0043] Oliver MJ Guo LN Alexander DC Ryals JA Wone BWM Cushman J 2011 A sister group contrast using untargeted global metabolomic analysis delineates the biochemical regulation underlying desiccation tolerance in *Sporobolus stapfianus* . The Plant Cell 23, 1231––1248 21467579 10.1105/tpc.110.082800 PMC3101564

[CIT0044] Ooms J Leon-Kloosterziel KM Bartels D Koornneef M Karssen CM 1993 Acquisition of desiccation tolerance and longevity in seeds of *Arabidopsis thaliana* . Plant Physiology 102, 1185––1191 12231895 10.1104/pp.102.4.1185 PMC158904

[CIT0045] Panza V Distéfano AJ Carjuzaa P Láinez V Del Vas M Maldonado S 2007 Detection of dehydrin-like proteins in embryos and endosperm of mature *Euterpe edulis* seeds. Protoplasma 231, 1––5 17602273 10.1007/s00709-007-0248-9

[CIT0046] Parcy F Valon C Kohara A Misera S Giraudat J 1997 The ABSCISIC ACID-INSENSITIVE3, FUSCA3, and LEAFY COTYLEDON1 loci act in concert to control multiple aspects of *Arabidopsis* seed development. The Plant Cell 9, 1265––1277 9286105 10.1105/tpc.9.8.1265 PMC156996

[CIT0047] Phillips JR Fischer E Baron M van den Dries N Facchinelli F Kutzer M Rahmanzadeh R Remus D Bartels D 2008 *Lindernia brevidens*: a novel desiccation-tolerant vascular plant, endemic to ancient tropical rainforests. The Plant Journal 54, 938––948 18346195 10.1111/j.1365-313X.2008.03478.x

[CIT0048] Reeves WM Lynch TJ Mobin R Finkelstein RR 2011 Direct targets of the transcription factors ABA-insensitive(ABI)4 and ABI5 reveal synergistic action by ABI4 and several bZIP ABA response factors. Plant Molecular Biology 75, 347––363 21243515 10.1007/s11103-011-9733-9 PMC3044226

[CIT0049] Schliesky S Gowik U Weber APM Bräutigam A 2012 RNA-seq assembly—are we there yet? Frontiers in Plant Systems Biology 3, 220 10.3389/fpls.2012.00220 PMC3457010 23056003

[CIT0050] Šunderlíková V Salaj J Kopecky D Salaj T Wilhem E Matušíková I 2009 Dehydrin genes and their expression in recalcitrant oak (*Quercus robur*) embryos. Plant Cell Reports 28, 1011––1021 19466427 10.1007/s00299-009-0710-6

[CIT0051] Tiedemann J Rutten T Mönke G 2008 Dissection of a complex seed phenotype: novel insights of FUSCA3 regulated developmental processes. Developmental Biology 317, 1––12 18343361 10.1016/j.ydbio.2008.01.034

[CIT0052] To A Valon C Savino G Guilleminot J Devic M Giraudat J Parcy F 2006 A network of local and redundant gene regulation governs Arabidopsis seed maturation. The Plant Cell 18, 1642––1651 16731585 10.1105/tpc.105.039925 PMC1488918

[CIT0053] Tolleter D Jaquinod M Mangavel C Passirani C Saulnier P Manon S Teyssier E Payet N Avelange-Macherel MH Macherel D 2007 Structure and function of a mitochondrial late embryogenesis abundant protein are revealed by desiccation. The Plant Cell 19, 1580––1589 17526751 10.1105/tpc.107.050104 PMC1913742

[CIT0054] Tunnacliffe A Wise MJ 2007 The continuing conundrum of the LEA proteins. Naturwissenschaften 94, 791––812 17479232 10.1007/s00114-007-0254-y

[CIT0055] Wall PK Leebens-Mack J Chanderbali AS 2009 Comparison of next generation sequencing technologies for transcriptome characterization. BMC Genomics 10, 347 19646272 10.1186/1471-2164-10-347 PMC2907694

[CIT0056] Walters C Berjak P Pammenter N Kennedy K Raven P 2013 Preservation of recalcitrant seeds. Science 339, 915––916 23430644 10.1126/science.1230935

[CIT0057] Wang F Perry SE 2013 Identification of direct targets of FUSCA3, a key regulator of Arabidopsis seed development. Plant Physiology 161, 1251––1264 23314941 10.1104/pp.112.212282 PMC3585594

[CIT0058] Zha HG Liu T Zhou JJ Sun H 2013 MS-desi, a desiccation-related protein in the floral nectar of the evergreen velvet bean (Mucuna sempervirens Hemsl): molecular identification and characterization. Planta 238, 77––89 23568404 10.1007/s00425-013-1876-2

[CIT0059] Zhulidov PA Bogdanova EA Shcheglov AS 2004 Simple cDNA normalization using kamchatka crab duplex-specific nuclease. Nucleic Acids Research 32, e37 14973331 10.1093/nar/gnh031 PMC373426

