# Supplementary material for: LEA polypeptide profiling of recalcitrant and orthodox legume seeds reveals ABI3-regulated LEA protein abundance linked to desiccation tolerance
Source: J Exp Bot. 2013 Sep 16;64(14):4559–73. doi: 10.1093/jxb/ert274 (PMC3808335; doi:10.1093/jxb/ert274)
Supplement: Supplementary Data [file supp_64_14_4559__index.html]

LEA polypeptide profiling of recalcitrant and orthodox legume seeds reveals ABI3-regulated LEA protein abundance linked to desiccation tolerance — Supplementary Data 

# LEA polypeptide profiling of recalcitrant and orthodox legume seeds reveals ABI3-regulated LEA protein abundance linked to desiccation tolerance

## Supplementary Data

Data files

**Files in this Data Supplement:**

- Supplementary Data - Supplementary Data
- Supplementary Data - Supplementary Data
- Supplementary Data - Supplementary Data
- Supplementary Data - Supplementary Data
- Supplementary Data - Supplementary Data
